# Vitamin B_12_ Metabolism during Pregnancy and in Embryonic Mouse Models

**DOI:** 10.3390/nu5093531

**Published:** 2013-09-10

**Authors:** Maira A. Moreno-Garcia, David S. Rosenblatt, Loydie A. Jerome-Majewska

**Affiliations:** 1 Department of Human Genetics, McGill University, 1205 Avenue Docteur Penfield, N5/13,Montreal, Quebec, Canada H3A 1B1; E-Mails: maira.moreno@mail.mcgill.ca (M.A.M.-G.); david.rosenblatt@mcgill.ca (D.S.R.); 2 Department of Pediatrics, McGill University, Montreal, Quebec, Canada H3H 1P3; 3 McGill University Health Centre, 4060 Ste. Catherine West, PT 420, Montreal, Quebec, Canada H3Z 2Z3

**Keywords:** cobalamin, mouse models, development, metabolism, vitamin B_12_

## Abstract

Vitamin B_12_ (cobalamin, Cbl) is required for cellular metabolism. It is an essential coenzyme in mammals for two reactions: the conversion of homocysteine to methionine by the enzyme methionine synthase and the conversion of methylmalonyl-CoA to succinyl-CoA by the enzyme methylmalonyl-CoA mutase. Symptoms of Cbl deficiency are hematological, neurological and cognitive, including megaloblastic anaemia, tingling and numbness of the extremities, gait abnormalities, visual disturbances, memory loss and dementia. During pregnancy Cbl is essential, presumably because of its role in DNA synthesis and methionine synthesis; however, there are conflicting studies regarding an association between early pregnancy loss and Cbl deficiency. We here review the literature about the requirement for Cbl during pregnancy, and summarized what is known of the expression pattern and function of genes required for Cbl metabolism in embryonic mouse models.

## 1. Introduction

Over the last two decades, there has been great interest of the role that nutritional factors such as folates and vitamin B_12_, play during embryonic development. One of the most prominent examples linking vitamins to development is the finding that the maternal periconceptional supplementation with folates can prevent occurrence and recurrence of neural tube defects [1,2]. However, even in the presence of folic acid fortification neural tube defects continue to occur [2,3], and thus interest in other interventions that could further reduce the prevalence of these disorders has increased. Vitamin B_12_, or cobalamin (Cbl), has also been identified as a crucial nutrient for foetal development [3,4]. The metabolisms of Cbl and folate are interrelated, with some of the biochemical and clinical effects of Cbl deficiency mediated by a functional deficiency of folate cofactors required for de novo nucleotide synthesis. A series of inborn errors of Cbl metabolism have been described, leading to identification of a number of genes encoding proteins involved in cellular Cbl metabolism. These inborn errors result in elevation of either homocysteine or methylmalonic acid, or both, in blood and urine, and can lead to human birth defects, including cardiovascular defects and facial dysmorphology.

Several studies have linked Cbl deficiency with increased risk of intrauterine growth retardation, abnormal embryo-foetal brain development, cleft palate and metabolic syndromes [3,5,6,7]. As deficiency of either folate or Cbl can result in similar biochemical effects, it has been suggested that a combined deficiency of Cbl and folate contribute to neural tube defects (NTD) and other birth defects. However, definitive proof of this hypothesis is lacking. In addition, although Cbl is also required for normal embryonic development in animal models, further studies are necessary to fully explain differences between findings in animal models and human patients with Cbl deficiency. In this manuscript we summarize what is known of the expression pattern of genes required for Cbl metabolism in the mouse model, and reviewed the literature in relation to the requirement for Cbl in human and mouse models.

## 2. Transport and Metabolism of Cobalamin

Cbl is required for cellular metabolism, and is obtained from the diet and found exclusively in animal products. Cbl contains the physiologically rare element, cobalt. The cobalt atom is surrounded by a corrin ring and upper and lower axial ligands. The lower axial ligand is an unusual ribonucleoside, dimethylbenzimidazole (DMB), which can be found in the base-on, or base-off conformation. In the base-on conformation the ligand is attached to the central cobalt atom, whereas in the base-off conformation the ligand is displaced from cobalt. The base-on and base-off conformations appear to play a role in the stability of different forms of Cbl, as well as their ability to bind to proteins [8].

Human uptake of Cbl is complex, requiring three Cbl binding proteins: intrinsic factor, haptocorrin and transcobalamin. Food-bound Cbl is released in the stomach where it is subsequently bound by haptocorrin (HC) [9]. Cbl is released from HC by pancreatic protease digestion in the jejunum, where it is bound by intrinsic factor (IF), to form an IF-Cbl complex. The IF-Cbl complex is bound by the cubam receptor, formed by two proteins amnionless and cubilin, which aids in the endocytosis of IF-Cbl into ileal enterocytes [10,11]. Dissociation of IF-Cbl complex occurs in the lysosome of enterocytes. Then, Cbl is exported into the portal circulation by the ATP-binding cassette (ABC) transporter ABCC1 (MRP1). Newly-absorbed Cbl appears in the portal circulation bound to transcobalamin (TC).

**Figure 1:**
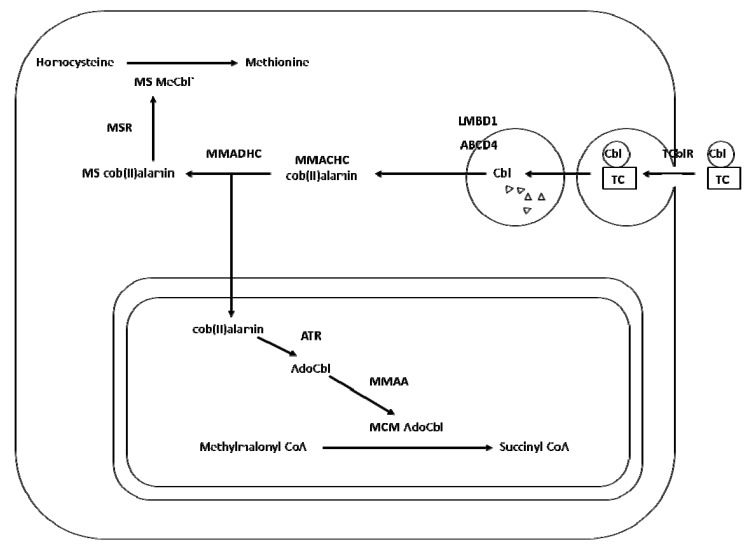
Intracellular processing of cobalamin. Cobalamin (Cbl) bound to transcobalamin (TC) is taken up by endocytosis and converted to methylcobalamin (MeCbl), which is required for activity of the cytoplasmic enzyme methionine synthase (MS) or adenosylcobalamin (AdoCbl), which is required for activity of the mitochondrial enzyme methylmalonylCoA mutase (MCM). TCblR, TC receptor; MSR, methionine synthase reductase.

In cells the TC-Cbl complex is recognized by the transcobalamin receptor CD 320 [12]. Once the TC-Cbl complex binds the TC receptor on the cell surface, a process of receptor-mediated endocytosis is triggered. The endocytic vesicle is directed to the lysosomal pathway and the lysosomal proteases of the compartment leads to a dissociation of the complex [13,14]. Free Cbl is translocated to the cytoplasm by two proteins: LMBD1 [15,16,17,18,19,20,21,22] and ABCD4 [23]. Once inside the cytoplasm, the central cobalt atom of Cbl must be reduced, and this process is thought to be aided by MMACHC and MMADHC proteins. MMACHC protein has a Cbl binding site and a TonB like domain [24]. MMACHC removes the upper axial ligand of dietary Cbl by reductive decyanation of CNCbl [25] or by dealkylation of either MeCbl or AdoCbl [26]. It has been postulated that MMACHC is the immediate downstream acceptor of the Cbl exiting the lysosome. MMACHC interacts with MMADHC, which presents Cbl to the cytosolic and mitochondrial proteins [27] acting as a branch point for Cbl delivery to the cytoplasm and mitochondria. However, the factors that determine the distribution of MMADHC between the cytoplasm and mitochondria remain unknown. Cbl can be converted to methylcobalamin (MeCbl), a coenzyme for methionine synthase (MS) in the cytosol, or 5-deoxyadenosylcobalamin (AdoCbl), a coenzyme for l-methylmalonyl-CoA mutase (MUT, MCM) in the mitochondria. In the cytosol, MS encoded by the *MTR* gene catalyzes the methylation of homocysteine to form methionine using 5-methyltetrahydrofolate as a methyl donor. In this reaction, MeCbl is the transient acceptor of the methyl group, with cycling of Cbl between its fully reduced cob(I)alamin and MeCbl forms. Methionine synthase reductase (MSR) encoded by the *MTRR* gene is required to maintain MS-bound Cbl in its active form by reducing partially-oxidized cob(II)alamin to MeCbl, with the transfer of a methyl group from adenosylmethionine. In the mitochondria, the MMAA protein binds to MCM and acts as a chaperone to prevent MCM inactivation [28,29]. The MMAB protein (ATP:cob(I)alamin adenosyltransferase) catalyzes the final step of AdoCbl biosynthesis [30,31]. In addition to converting the reduced form of Cbl into AdoCbl, the MMAB protein may also act as a chaperone ensuring AdoCbl’s delivery to its target, MCM [32]. In summary, Cbl is necessary for: (i) the remethylation of homocysteine and the production of methionine, the precursor of *S*-adeno-sylmethionine (SAM). SAM is the major donor of methyl groups for many substrates such as: DNA, RNA, histones and co-regulators of nuclear receptors that plays a key role in epigenetic and epigenomic mechanisms and (ii) the metabolism of branched chain amino acids, odd-chain fatty acids and cholesterol and catalyzes the reversible isomerization of l-methylmalonyl-CoA to l-succinyl-CoA, which is key to the breakdown of propionate as well as for replenishing the TCA cycle (Figure 1).

## 3. Cobalamin Deficiency, Polymorphism and Risk of Neural Tube Defects

Cbl deficiency, in association with folate deficiency is potentially a health problem during pregnancy. Human studies are numerous and the results are interesting although not conclusive. Low concentrations of Cbl in the mother’s serum have been linked with increased risk of neural tube defects, (NTD) in Irish, Canadian, Chinese, and Egyptian populations [33,34,35,36,37]. However, other studies have found no effect of Cbl status [38,39,40,41,42,43,44,45,46,47]. These results have been reviewed by Ray and Blom 2003, who identified a moderate association between low maternal serum Cbl levels and risk of NTD [48]. In amniotic fluid from neural tube defect pregnancies, Cbl levels have been measured, and in most cases have been lower in affected pregnancies compared to controls [39,49,50,51,52,53,54]. However, in mothers who previously had a child with NTD there was no difference in Cbl levels in amniotic fluid compared to controls [52].

Holotranscobalamin (holoTC; Cbl bound to TC), is the fraction of circulating Cbl that is available for tissue uptake. Decreased levels of holoTC were observed in the serum of mothers who had a child with NTD [55,56] or who were pregnant with a NTD fetus [35]. In addition, the levels of apo-haptocorrin and apo-TC were doubled and tripled respectively in amniotic fluid from pregnant women who had a child with NTD compared with control groups [55], suggesting that holoTC is a better reflector of Cbl status [57,58]. This is in contrast to the findings of AlAisari *et al.*, who showed that holoTC and total Cbl, alone and in combination, have almost equal diagnostic efficiency in screening/diagnosing Cbl status in patients investigated for Cbl deficiency [59]. The Cbl status of mothers with infants with cleft lip or palate has not been extensively analyzed. One study of serum Cbl in mothers who had delivered a child with cleft lip showed a significant decrease in Cbl levels compared to controls [60], but not all studies show the same results [61,62]. Elevated propionylcarnitine in infants is a marker of maternal Cbl deficiency when total blood propionylcarnitine concentrations were measured in newborns with orofacial clefts, the authors did not find any significant differences in the mean concentrations between newborns with clefts and controls, suggesting that the deficiency of Cbl seems not to be a risk factor for CLP [6].

Human studies also have shown a link between polymorphisms in genes involved in Cbl transport and metabolism with the risk of developing neural tube defects. A polymorphism (rs1907362) in the 27th intron of the *CUBN* gene has been linked with a decreased risk of spina bifida, and was related to increased serum Cbl levels [63]. A polymorphism in the *MTRR* gene (c.66A>G) has been associated with an increased risk of developing a NTD in offspring, with the GG or AG polymorphism [64,65,66,67,68,69]. In contrast, two studies appeared to demonstrate a protective effect of the G allele [70,71]. Other studies have not shown any effect on neural tube defect risk [49,72,73,74,75] or risk of other birth defects [49,72,76]. Polymorphisms in the *MTR* gene (2756A>G; 2758C>G) result in various health problems before birth [64,69,70,77]. However, no association has been found between these polymorphisms and increased maternal risk for giving birth to children with NTD [78]. The 776C>G polymorphism in the *TCN2* gene has been linked with increased risk of NTD-affected pregnancy in Irish and Italian populations [79,80] and the association of the G allele with spina bifida has been reported [66]. Significant over transmission of the *TCN2* C allele in offspring with cleft lip with or without cleft palate has been observed [81].

## 4. Expression Pattern of Cbl Genes during Mouse Organogenesis

In the hope of identifying the etiology of developmental abnormalities and possible maternal contribution to the phenotypes associated with Cbl metabolism, we, and others, have evaluated the expression pattern of the genes associated with Cbl metabolism (*Mmaa*, *Mmab*, *Mmachc*, *Mmadhc*, *Mtr*, *Mtrr*, *Lmbrd1*, *Abcd4* and *Mut*) by *in* *situ* hybridization and immunohistochemistry in wild type mouse placentas and embryos [82,83,84,85,86,87,88].

The expression patterns of *Mtr*, *Mmaa*, *Mmab* and *Mut* were analyzed at several stages of placental development. *Mtr* was highly expressed in syncytiotrophoblast cells of the labyrinth (functional) layer and trophoblast giant cells of the mouse placenta. The strongest expression of *Mtr* was between embryonic day (E) E9.5–E12.5. At these stages, *Mtr* was expressed in the S-TGC that lines the maternal sinuses and provides endocrine support for the pregnancy. *Mtr* was also highly expressed in parietal trophoblast giant cells (P-TGCs) between E8.5–E10.5. The P-TGC serves to separate the embryonic portion of the placenta from the maternal decidua. In addition, *Mtr* was highly expressed in very invasive trophoblast giant cells such as canal trophoblast giant cells (C-TGCs) that lines the maternal vessels which bring blood to the placenta, and in glycogen trophoblast (GlyT) cells at E12.5, E15.5 and E18.5 [82]. We reported broad expression of *Mmaa*, and *Mmab* in the labyrinth layer, giant cells and spongiotrophoblast cells of placentas from E10.5 embryos. In contrast, we did not find high expression of *Mut* in the placenta [85]. The expression patterns observed in the placenta suggest that *Mtr*, *Mmaa*, and *Mmab* may be important for normal Cbl metabolism in the placenta. It will be interesting to determine if proteins required for Cbl uptake are also expressed in the placenta as this might suggest that Cbl is not only transported across the placental barrier but is utilized in this organ.

The expression patterns of *Abcd4*, *Mmachc*, *Mmadhc*, *Mtrr*, *Mtr*, *Mmaa*, *Mmab* and *Mut* have been reported during different stages of mouse development. At E14.5, the only stage analyzed, *Abcd4* was expressed in the central nervous system, telecephalon, and neocortex [88]. Expression of *Abcd4* was also detected in the mantle layer of the cerebral cortex [83]. Intriguingly, expression of this gene has not been reported outside of the nervous system.

*Mmachc* and *Mmadhc* expression was analyzed at E11.5. Unlike *Abcd4*, these genes were broadly expressed in most organs. *Mmachc* and *Mmadhc* were both expressed in the dorsal root ganglia, notochord, head mesenchyme, developing central nervous system (CNS), developing neck region (pharyngeal endoderm and the esophagus), mesonephric kidney, stomach, bulbus cortis, heart, lungs and endothelium of blood vessels. However, in the developing heart, lung, and stomach, *Mmachc* expression was cell type specific being limited to the cushions of the heart, right ventricle, and endoderm of the lung and stomach [86]. This data suggest that although products of the two genes are proposed to interact, they may not do so in every single cell type.

*Mtrr* expression was analyzed in mice carrying a gene-trap allele that resulted in knock-in of LacZ into the locus. β-Galactosidase activity at E9.5 and E10.5 was used as a surrogate for gene expression. β-Galactosidase activity was found throughout the embryo with higher activity detected in the optic eminence, forebrain (telencephalon and diencephalon), midbrain, 2nd and 4th rhombomeres of the hindbrain, notochord and the neural tube. High β-galactosidase activity was also detected within the first brachial arch, splanchnic mesoderm, hindgut, and foregut. The observed sites of β-galactosidase activity at these stages suggest that *Mtrr* is broadly expressed during embryogenesis [84]. At E14.5, *Mtrr* expression was also reported in the developing brain, in the upper and lower cortical plate, cerebral cortex subventricular zone and ventricular layer [87].

Visel *et al.*, reported expression of *Mtr* at E14.5 in developing embryos [82,88]. *Mtr* expression was expressed in the forelimb, hindlimb, integumental system, and skin. Moderate *Mtr* expression was found in the nervous system, central nervous system, telencephalon, cerebral cortex, midbrain, inferior colliculus, spinal cord and tail. Weak *Mtr* expression was observed in the hippocampus, basal ganglia, neocortex, corpus striatum, choroid plexus, peripheral nervous system, cranial ganglion, dorsal root ganglion, eye, retina, alimentary system, tooth, renal-urinary system, reproductive system and genital tubercle. Thus, similar to *Mtrr*, *Mtr* was broadly expressed in developing embryos.

We also reported the expression pattern of *Mmaa*, *Mmab* and *Mut* at E10.5–E12.5 [85]. At E10.5 we found higher expression of *Mmaa*, *Mmab* and *Mut* in the heart, branchial arches, and neural tube. By E11.5, we found co-expression of these genes in the dorsal root ganglion, developing heart, liver and tissue-specific expression pattern of these genes in head mesechyme and head endothelial vessels. However, at this stage, expression of *Mmaa* and *Mmab* was more robust than that of *Mut.* At E12.5, all three genes were expressed in head mesenchyme, blood vessels, heart and kidney. *Mmaa* and *Mmab* expression was tissue-specific in organs such as lung, gut, liver and urogenital sinus. In lung, *Mmaa* was most highly expressed in the endothelial cells of vessels and was not observed in the lung buds. In midgut, low levels of *Mmab* were detected in the mesenchyme but not in the gut endoderm. In the region of the developing urogenital sinus and rectum, we detected expression of *Mmaa* throughout of the urogenital sinus whereas *Mmab* was highly expressed in both the urogenital sinus and rectum, and *Mut* was also expressed in the urogenital sinus and rectum [85].

Although incomplete, the studies published to date suggest that genes associated with Cbl metabolism are not all ubiquitously expressed (Table 1). However, the expression pattern of these genes overlaps in the neural tube, dorsal root ganglion and heart, except for *Mtr* which was only analyzed at E14.5. These data provide evidence that Cbl may be necessary for normal neural tube, dorsal root ganglion, heart development and function during mouse organogenesis. These data also support a possible developmental origin for clinical features seen in Cbl deficiency patients such as neural tube defects, heart malformations and neurological disorders. It is clear that in addition to the expression pattern of the genes associated with *cblF* and *cblJ*, the expression pattern of all of these genes needs to be analyzed at similar stages in order to generate a complete atlas of where genes in the Cbl pathway are expressed during embryogenesis and the organs in which their Cbl metabolism may be critical for normal morphogenesis.

**Table 1:** Summary of the main sites of expression of genes in vitamin B12 absorption during mouse embryogenesis from E9.5–E14.5. **1**: only analyzed at E14.5; **2**: only analyzed at E11.5; **3**: only analyzed at E11.5, * ubiquitous expression; **4**: analyzed at E9.5–E10.5 and E14.5, * ubiquitous expression at E9.5–E10.5; **5**: only analyzed at E14.5; **6**: analyzed at E10.5–E12.5; * ubiquitous expression at E10.5; **7**: analyzed at E10.5–E12.5, * ubiquitous expression at E10.5; **8**: analyzed at E10.5–E12.5, * ubiquitous expression at E12.5. Dorsal root ganglion (drg).

		Genes							
Organs		*Abcd4* 1	*Mmachc* 2	*Mmadhc* 3 *	*Mtrr* 4 *	*Mtr* 5	*Mmaa* 6 *	*Mmab* 7 *	*Mut* 8 *
**Branchial** **arches**					+		+	+	+
	Mouth						+	+	+
	Nose						+	+	+
	Palate						+	+	+
	nasal cavity		+	+			+	+	+
	Tongue		+	+			+	+	+
	Teeth					+			
**Head**									
	Head mesenchyme		+	+			+	+	
	Endothelial vessel of head						+	+	+
**Neural crest cells**									
	Drg		+	+	+	+	+	+	+
**Neural tube**			+	+	+		+	+	+
	Brain	+		+		+			
	spinal cord	+	+	+		+			
	Forebrain	+			+	+			
	Midbrain				+	+			
	Hindbrain				+				
**Rathkete’s Pouch**							+	+	
	Pituitary						+	+	+
**Eye and ear**									
	Eye					+			
	Retina					+			
**Somite**				+					
	Intersomitic blood vessels						+	+	
	Condensing somites								+
**Notochord**			+	+	+				
**Heart**			+	+	+		+	+	+
	Atria		+						
	Bulbus cordi		+						
	Endothelium		+	+					
**Gut**								+	+
	Liver		+	+	+		+	+	+
	Esphagus		+	+	+				
	Stomach		+	+	+				
	Pancreas				+				
	Intestine		+		+				
	Rectum				+				
	Anus				+				
**Limbs**									
	Forelimb					+			
	Hindlimb					+			
**Urogenital sinus**						+			
	Kidneys		+				+	+	+
	Ureters						+		
	Bladder						+	+	+
	Urethra								
**Genital sinus**						+			
**Integumental **						+			
	Skin					+			
**Respiratory system**									
	Lungs		+	+	+		+	+	+

## 5. Mouse Models

### 5.1. Cobalamin Absorption

Amnionless and cubilin help in absortion of Cbl from the blood stream into the epithelial cells of the distal ileum [89]. The link between these proteins and a variety of developmental defects has been shown. The importance of this complex for normal fetal development was initially noted when antibodies that blocked their activities in yolk sac endoderm resulted in severe developmental abnormalities. Injection into rodents of antibodies raised against rat kidney, placenta and yolk sac resulted in decreased fetal weight, increased resorption rates, and increase incidence of cardiovascular defects, urogenital malformations, orofacial clefts, hydrocephalus, exencephaly and anencephaly. Later studies indicated that the antibodies recognized the Cbl intrisic-factor complex [90].

The human amnionless gene contains seven transcription start sites, two of which are positioned after exon 4 [91]. Mutations in exons 1–4 of the human amnionless gene cause megaloblastic anemia, due to malabsorption of Cbl [92]. Interestingly, mouse embryos lacking the amnionless gene product do not survive past the 10th day of gestation, have a poorly developed amnion, and an absence of trunk mesoderm [93]. Since human patients have been reported with mutations in the first seven exons of amnionless, it is postulated that the *C*-terminus of the protein is required for proper embryonic development [91]. This case is further supported by recent reports of patients with truncating mutations in *Amn* [94,95].

In mice, cubilin deficiency results in embryonic developmental defects that are smilar to those found in *Amn* embryos. Homozygous cubilin knockout embryos are developmentally delayed with absence of somite formation and abnormal endodermal function [96]. Human cubilin is a large peripheral membrane protein that contains eight EGF-like and 27 CUB domains [97]. Mutations in cubilin have been associated with impaired ligand binding of IF-Cbl, which results in megaloblastic anemia [98]. Recently, a renal-biopsy specimen from a patient that was diagnosed with cubulin deficiency and a homozygous mutation in cubilin showed no immunologic reaction for cubulin and abnormal cytoplasmic and vesicular distribution of amnionless with no developmental defects [99], suggesting that cubilin may not be required for normal human development. In addition, although cubilin was initially described as a receptor for the IF-Cbl complex, it was also found to be involved in endocytosis of high-density lipoproteins and a number of other ligands [100]. Therefore, the variety of roles played by cubilin in humans, and the difference in phenotypes of rodents and humans with cubilin deficiency, indicate that this protein may play differing roles between the two species.

### 5.2. Cobalamin and Folic Acid Pathways

Folic acid and Cbl deficiency is a risk factor for pathogenic conditions such as neural tube, limb, cardiac and jaw defects in fetal development. The importance of folic acid in preventing birth defects, and particularly neural tube defects during embryonic development has been widely reported and modelled in the mouse [101]. In rat myocardium, Cbl deficiency during gestation and lactation results in postnatal growth retardation, myocardium hypertrophy (cardiomyocyte enlargement) lipid droplets, and decreases respiratory activity of complexes I and II with disturbed mitochondrial alignment, suggesting that Cbl deficiency may induce perinatal cardiomyopathies [102].

Folic acid and Cbl pathways are connected at the step where homocysteine is converted into methionine. In the cell, Cbl acts as a cofactor to MS (*MTR*), which catalyzes the re-methylation of homocysteine to methionine and the concurrent de-methylation of 5-methyltetrahydrofolate (5-Me-THF) to tetrahydrofolate (THF).

In mice, complete loss of *Mthfd1*, the gene which encodes the protein that catalyzes the formation and intercorvertion of folate-activated one carbon groups required for nucleotide biosynthesis and cellular methylation, results in early embryonic lethality during development. However, mice heterozygous for a *Mthfd1* null allele were viable and exhibited impaired cellular methylation with a 50% decrease of MTHFD1 proteins levels. In addition, maternal *Mthfd1* disruption contributed to abnormal embryonic development with growth retriction, irregular neuroepithelial organization, and torqued body symmetry at E11.5 without any neural tubes defects [103].

Homozygosity for loss of function mutations in either *Mtr* or *Mtrr*—the genes mutated in *cblG* and *cblE* patients, respectively—is embryonic lethal in mice [84,104]. Mouse embryos with MS deficiency can implant but die soon after. Whereas mice heterozygous for the *Ms* null allele have a 60% reduction in MS activity, are viable and survive to adulthood [104]; hypomorphs of *Mtrr* exhibit metabolic derangement of methionine and folate metabolism with increased plasma homocyst(e)ine, increased tissue methyltetrahydrofolate, and decreased plasma methionine with no change in AdoMet/AdoHcy ratio in most tissues [84]. In E14.5, embryos *Mtrr* deficiency results in congenital heart defects such as myocardial hypoplasia and higher incidence of ventricular septal defects (VSD) and reduced embryonic length. In mothers, *Mtrr* deficiency results in adverse reproductive outcomes with more resorptions, more delayed embryos and smaller placenta [105].

Homozygosity for loss of function mutations in 5,10-methylenetetrahydrofolate reductase (*Mthfr*) results in reduced survival at two weeks of age with a series of neurological complications: motor and gait abnormalities or delayed development [106], cerebellar abnormalities with effects on granule cell development, neuronal organisation [107], and neurobiological changes in the hippocampus [108], suggesting that *Mthfr* is important for proper neuronal development in mice. Similar to humans, maternal *Mthfr* deficiency and low dietary folate in mice lead to adverse reproductive outcomes such as increased incidence of pregnancy loss, fetal growth retardation and congenital heart defects [109].

### 5.3. Cobalamin and Methylmalonyl-CoA Mutase (MCM)

Mouse models with mutations in *Mmaa*, *Mmab*, *Mmachc*, *Mmadhc* or *Lmbrd1* have yet to be described. Although, MCM is not required during embryogenesis, targeted deletion of *Mut* results in perinatal lethality [108]. Mutations affecting MCM, which participates in the catabolism of odd-chain fatty acids, some branched amino acids and cholesterol, is the mouse model that has been widely used to study defects in one-carbon metabolism. Mouse pups with no functional copy of the *Mut* gene (*Mut*^−/−^) are normal at birth but show abnormal breathing, reduced movement, reduced suckling, and die within 24 h of birth [110] with elevated levels of methylmalonic and methylcitric acids. However, when the *Mut* mutation was placed on a mixed Fbv/n genetic background, a number of *Mut*^−^^/^^−^ mice survived and exhibited large mitochondria in kidney, liver and pancreas, a phenotype that was later confirmed in human liver samples from *mut* patients [111].

Neonatal lethality of *Mut^−−/−−^* mice was rescued by virus-mediated gene therapy, through intrahepatic injections of adenovirus carrying the *Mut* gene (Ad-Mut-GFP), (rAAV8), (rAAV9) [112,113,114]. Survival of homozygous mutant pups was monitored for longer than one year, and although expression of the transgene decreased over time, the mice remained indistinguishable from wild type littermates in size and activity levels. Treated *Mut*^−−/−−^ mice lived beyond one year of age, had improved growth, lower plasma methylmalonic acid levels, and an increased capacity to oxidize propionate *in vivo* [115], thus illustrating the power of mouse models and the need for additional models in this system.

Transgenic mice carrying an intact human *MUT* locus also have been produced. Transgenic mice were crossed with heterozygous knockout *Mut* mice to generate mice hemizygous for the human transgene on a homozygous knockout background. Partial rescue of the uniformly neonatal lethality in homozygous knockout mice was observed. These rescued mice were significantly smaller than mice with the wild type *Mut* gene and exhibited elevated methylmalonic acid levels in urine, plasma, kidney, liver and brain tissue. The human transgene was expressed at higher levels in the kidney followed closely by brain and liver as compared to the wild type mice [116], confirming that the human and mouse proteins are almost completely interchangeable.

In addition, a mouse model that contains a human methylmalonyl-CoA mutase locus carrying a stop codon mutation identified in a patient with *Mut* MMA was generated. The transgene was found to be intact in the mouse model, with seven copies integrated at a single site in chromosome 3. The phenotype of the hemizygous mouse was unchanged until crossed against a methylmalonyl-CoA mutase knockout mouse. Pups with no endogenous mouse methylmalonyl-CoA mutase and one copy of the transgene became ill and died within 24 h. This severe phenotype was partially rescued by the addition of a transgene carrying two copies of the normal human methylmalonyl-CoA mutase locus [117]. In addition, the “humanized” mice mimicked the key features of the human MMA disorder allowing the authors to use genetic therapies such as fetal progenitor cell transplantation in the liver, bone marrow and spleen to treat this disorder [117].

Finally, administration of folinic acid and Cbl together has been shown to be protective against a number of malformations including neural tube defects, branchial arch abnormalities and cardiac defects caused by ethanol exposure during mouse pregnancies, whereas, Cbl or folinic acid alone did not have any significant protective effect [118]. Recently, Cbl was shown to support palate fusion in the presence of concentrations of dexamethasone, which normally impaired fusion of murine palates in organ culture [119]. *In vivo*, Cbl in the presence of dexamethasone restored proliferation of the mesenchymal cells of the palate via increased expression of a growth factor—Fgf10—which is normally involved in craniofacial development [120], and thus, suggesting that Cbl may indirectly regulate the levels of growth factors essential for normal organogenesis.

## 6. Conclusions

The genes involved in the vitamin B_12_ metabolism are not ubiquitously expressed during embryogenesis suggesting that the proteins encoded by these genes may not interact throughout organogenesis. These findings lead us to postulate that a subset of the genes involved in vitamin B12 metabolism may have “moonlighting” functions. Thus, it is possible that developmental phenotypes in patients with mutations in these genes are due to (a) abnormal vitamin B_12_ metabolism and/or (b) some other unknown requirement for the proteins encoded by genes in the vitamin B_12_ pathway. In the future, mouse models of *Mmachc*, *Mmadhc*, *Lmbrd1* and *Abcd4* deficiency may shed insight into the roles of these proteins during development and explain the etiology of developmental phenotypes in a subset of patients. Proper understanding of the role of Cbl in embryonic development will also determine whether Cbl supplementation should be used in conjunction with folic acid supplementation in order to further prevent the occurrence of neural tube and heart birth defects during pregnancy.
